# *Cgr1*, a ripe rot resistance QTL in *Vitis amurensis* ‘Shuang Hong’ grapevine

**DOI:** 10.1038/s41438-019-0148-0

**Published:** 2019-06-01

**Authors:** Peining Fu, Quanyou Tian, Gongti Lai, Rongfang Li, Shiren Song, Jiang Lu

**Affiliations:** 1 Viticulture and Enology Program, College of Food Science and Nutritional Engineering, China Agricultural University, Beijing, 100083 China; 2 Center for Viticulture and Enology, School of Agriculture and Biology, Shanghai Jiao Tong University, Shanghai, 200240 China

**Keywords:** Agricultural genetics, Plant hybridization

## Abstract

Ripe rot is a serious grapevine disease in *Vitis* L. and *Muscadinia* (Planch.) Small. However, resistance to this disease has been reported in some oriental *Vitis* species. To identify resistance-related Quantitative Trait Loci (QTLs) from the Chinese grape species *V. amurensis*, an F_1_ population of *V. vinifera* ‘Cabernet Sauvignon’ × *V. amurensis* ‘Shuang Hong’ was used to map the ripe rot resistance loci expected in ‘Shuang Hong’ grape. A total of 7598 single nucleotide polymorphisms (SNPs) between the parents were identified in our previous study, and 934 SNPs were selected for genetic map construction. These SNPs are distributed across the 19 chromosomes covering a total of 1665.31 cM in length, with an average of 1.81 cM between markers. Ripe rot resistance phenotypes among the hybrids were evaluated in vitro using excised leaves for three consecutive years from 2016 to 2018; a continuous variation was found among the F_1_ hybrids, and the Pearson correlation coefficients of the phenotypes scored in all three years were significant at the 0.01 level. Notably, the first QTL reported for resistance to grape ripe rot disease, named *Cgr1*, was identified on chromosome 14 of ‘Shuang Hong’ grapevine. *Cgr1* could explain up to 19.90% of the phenotypic variance. In addition, a SNP named ‘np19345’ was identified as a molecular marker closely linked to the peak of *Cgr1* and has the potential to be developed as a marker for the *Cgr1* resistance haplotype.

## Grapevine: Genomic region linked to fungal resistance in Chinese varietal

Researchers in China have discovered the first region of the grapevine genome known to confer resistance to ripe rot, a fruit disease caused by fungal pathogens. A team led by Shiren Song and Jiang Lu from Shanghai Jiao Tong University crossbred a European grape species that’s susceptible to ripe rot with a Chinese grape species that’s resistant to the disease. They inoculated the progeny’s leaves with the fungus and searched for genetic differences that could explain variation in rot symptoms. In this way, the researchers identified a segment of chromosome 14 from the Chinese Amur grape, *Vitis amurensis* ‘Shuang Hong’, that accounted for about 20% of difference in resistance capacity between the two grape species. This information could help breeders select more rot-resistant grapevines in the future.

## Introduction

Grape ripe rot disease, caused by *Colletotrichum gloeosporioides* (Penzig) Penz. & Sacc^1^. or *Colletotrichum acutatum*^2^, results in sunken necrotic lesions on stems, flowers, leaves, and fruit clusters^3^. In most grapevine planting regions of China, especially in southern China with rainy and humid veraison and maturity periods, *C. gloeosporioides* has become the main causal agent of grape ripe rot^4^.

Fungicide application is the most effective way to control grape ripe rot^5^. Because veraison and maturity are the main periods for *C. gloeosporioides* infection, application of fungicides is indispensable. The period between fungicide spraying and fruit ripening is too short to avoid the risk of introducing fungicide residues in berry and products derived from them (must, wine, and raisin). Garcia-Cazorla et al.^6^ detected fungicide residues in berry, must, and wine after monthly spraying of fungicides, and the amount of residues in berry was higher than those in must and wine. In addition, fungicide spraying is labor-intensive, costly, and damaging to the environment. Therefore, developing ripe rot-resistant varieties with high fruit quality would be beneficial to the grape industry.

Ripe rot, a serious disease in grapevine, has been reported in many species of *Vitis* L.^4^ and *Muscadinia* (Planch.) Small^7^. Li et al.^8^ evaluated ripe rot resistance in 56 accessions of Chinese wild *Vitis* species and found all of them to be resistant to ripe rot disease. Among them, there were 8 *V. amurensis* accessions, including ‘Shuang You’. ‘Shuang Hong’, which shares a common parent, ‘Shuang Qing’, with ‘Shuang You’, was used to investigate the genetics of ripe rot resistance in *V. amurensis* in this study.

Genetic mapping is commonly used for identifying genetic loci of interest. In grapevine, many genetic maps have been constructed using first- and second-generation markers, such as restricted fragment length polymorphisms (RFLP)^9,10^, amplified fragment length polymorphisms (AFLP)^10,11^, random amplification of polymorphic DNA (RAPD)^9,11^, and simple sequence repeats (SSR)^12,13^. However, the intervening distances among these markers are usually too long to fine-map the candidate genes. Single nucleotide polymorphisms (SNPs), as third-generation molecular markers, are the most abundant markers in grapevine and very useful for fine-mapping^14–16^. With the development of a library pooling strategy and high-throughput DNA sequencing technology^17^, SNP calling has developed into a time- and cost-saving marker technology. In the present study, we used a “genotyping-by-sequencing” strategy to identify SNPs for constructing genetic maps in a population of *V. vinifera* ‘Cabernet Sauvignon’ and *V. amurensis* ‘Shuang Hong’ to identify ripe rot resistance Quantitative Trait Loci (QTLs) in the latter.

## Results

### Construction of genetic map

The cross-pollination (CP) model of JoinMap 4.0 was used to construct the genetic maps. Markers were segregated into different linkage groups by the ‘independence LOD’ function, and a LOD score of ‘7’ was set as the threshold for determining whether loci were linked or not. Subsequently, the Kosambi function was used to calculate the distance between markers.

The map of the female parent, ‘CS’, contained 559 SNPs across 19 chromosomes (Chrs) covering 1506.57 cM, with an average marker distance of 2.93 cM (Table 1). The number of SNPs on the Chrs ranged from 13 (Chr09) to 43 (Chr18), with an average of 29.4 markers per Chr. The map of the male parent, ‘SH’, contained 511 SNPs covering 1440.14 cM across 19 Chrs, with an average marker interval of 3.36 cM (Table 1). The number of SNPs ranged from 12 (Chr18) to 42 (Chr13), with an average of 26.9 markers per Chr.

**Table 1:** Data of linkage groups in the maternal parent ‘Cabernet Sauvignon,’ paternal parent ‘Shuang Hong,’ and integrated maps

LGs	Map of female parent ‘Cabernet Sauvignon’			Map of male parent ‘Shuang Hong’			Integrated map		
	Covered length (cM)	No. of markers	Average distance (cM)	Covered length (cM)	No. of markers	Average distance (cM)	Covered length (cM)	No. of markers	Average distance (cM)
01	98.12	33	3.07	103.93	17	6.50	108.15	48	2.30
02	77.99	34	2.36	69.52	27	2.67	74.90	48	1.59
03	75.31	31	2.51	75.75	25	3.16	80.71	49	1.68
04	95.65	33	2.99	76.00	20	4.00	94.31	50	1.92
05	84.27	29	3.01	73.59	34	2.23	93.86	50	1.92
06	89.55	36	2.56	88.60	17	5.54	90.43	49	1.88
07	71.50	28	2.65	108.22	29	3.87	116.02	50	2.37
08	91.63	37	2.55	85.65	26	3.43	95.67	49	1.99
09	44.31	13	3.69	61.64	39	1.62	82.09	48	1.75
10	78.70	24	3.42	52.65	34	1.60	100.25	50	2.05
11	69.70	33	2.18	84.88	26	3.40	71.83	50	1.47
12	81.19	30	2.80	74.88	27	2.88	88.22	50	1.80
13	83.82	25	3.49	100.63	42	2.45	101.81	50	2.08
14	78.47	27	3.02	73.15	27	2.81	78.88	50	1.61
15	96.65	28	3.58	70.91	26	2.84	94.55	50	1.93
16	37.13	20	1.95	34.86	29	1.25	41.95	43	1.00
17	81.90	40	2.10	81.88	14	6.30	87.51	50	1.79
18	94.56	43	2.25	64.78	12	5.89	91.99	50	1.88
19	76.12	15	5.44	58.62	40	1.50	72.16	50	1.47
Total	1506.57	559	2.93	1440.14	511	3.36	1665.31	934	1.81

**Fig. 1:**
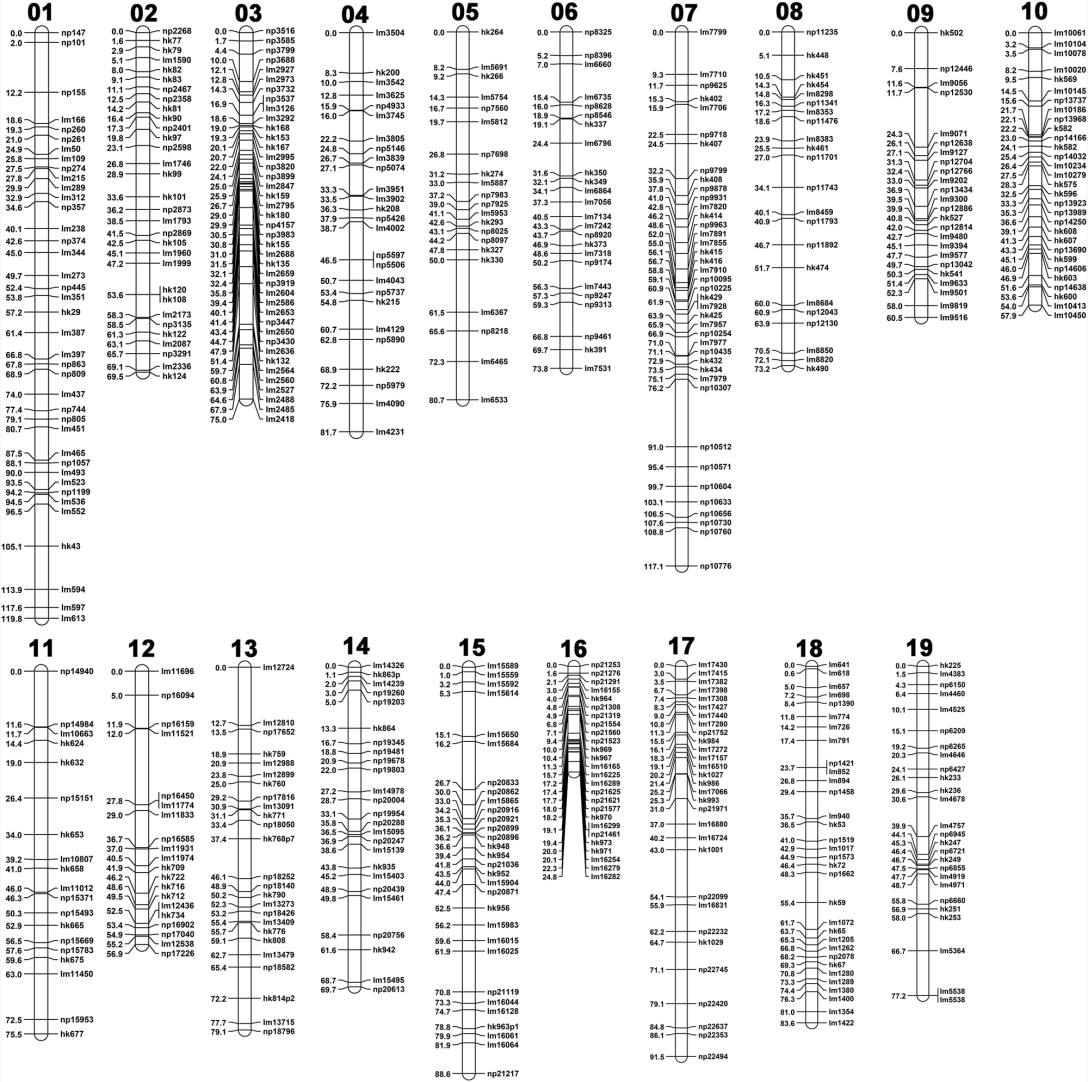
Integrated genetic map of ‘Cabernet Sauvignon’ × ‘Shuang Hong’

After construction of maps for the parents, the ‘Combine Groups for Map Integration’ function was used to construct the consensus map. This map contained 934 SNPs and covered 1665.31 cM on 19 Chrs. The average marker interval was 1.81 cM (Table 1; Fig. 1). The average number of SNPs per chromosome was 49.2. The genetic distance of the linkage groups ranged from 41.95 (Chr16) to 116.02 cM (Chr07), with an average length of 87.65 cM per Chr.

### Phenotypic analysis of parents and progeny

**Fig. 2:**
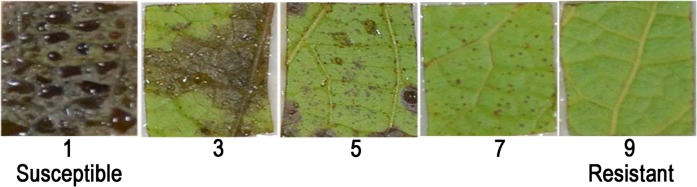
Leaf symptoms of ripe rot disease representing different levels of resistance/susceptibility

**Fig. 3:**
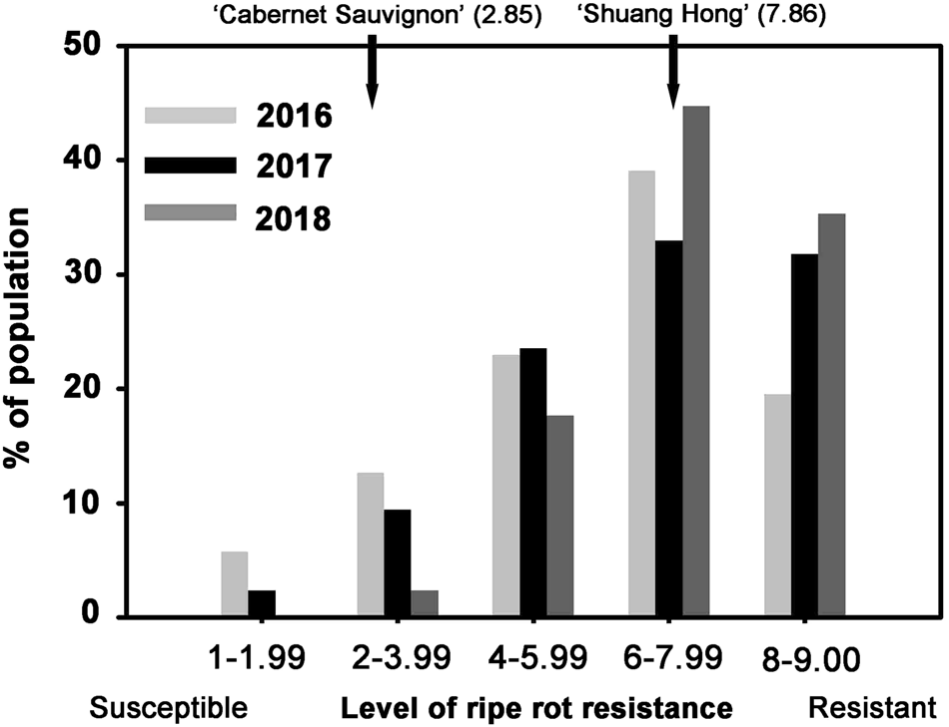
Distribution of ripe rot phenotype scores among 91 hybrids of ‘Cabernet Sauvignon’ × ‘Shuang Hong’

Resistance to *C. gloeosporioides* was scored from 1 (most susceptible) to 9 (most resistant) (Fig. 2). The average scores for the parents during the three-year period (2016–2018) were 2.85 for CS and 7.86 for SH (Fig. 3). Resistance to *C. gloeosporioides* displayed continuous variation in the CS × SH hybrids and appeared to be a quantitative trait (Fig. 3). Most of the progeny fell into the resistance range of 6–7.99 (32.9–44.7%), followed by 8–9.00 (19.–35.3%). Some individuals showed transgressive segregation (Fig. 3). The Pearson correlation coefficient of the *C. gloeosporioides* resistance scores between 2016 and 2017 was 0.41, that between 2017 and 2018 was 0.40, and that between 2016 and 2018 was 0.28; all were significantly correlated at the 0.01 level (Table 2).

**Table 2:** Pearson correlation coefficients of ripe rot scores in 3 years

Pearson correlation	2016	2017	2018
2016	1.00	0.41^a^	0.28^a^
2017		1.00	0.40^a^
2018			1.00

^a^The correlation is significant at the 0.01 level

### Nonparametric test

The rank-sum test, performed using the Kruskal–Wallis algorithm, identified 21 markers on Chr14 that cosegregated along with *C. gloeosporioides* resistance in three years (Table 3). Four of these 21 markers (np19345, np19481, np19678, and np19803) were identified in all three years, among which np19345 was the marker that cosegregated most significantly with *C. gloeosporioides* resistance.

**Table 3:** Identification of markers cosegregated with resistance to *C. gloeosporioides* using the Kruskal–Wallis algorithm

Locus	Chromosome	Df^a^	Significance level		
			2016	2017	2018
np19260	14	1	****	**	–
np19203	14	1	***	*	–
lm14326	14	1	–	–	*
lm14239	14	1	–	–	**
hk863	14	1	–	–	*
np19345	14	1	******	*****	***
np19481	14	1	******	****	**
np19678	14	1	*****	****	*
np19803	14	1	*****	*****	**
np20004	14	1	*****	******	–
np19954	14	1	*****	****	–
np20288	14	1	****	**	–
np20247	14	1	****	**	–
lm15095	14	1	–	–	*
lm15139	14	1	–	–	*
lm15403	14	1	–	*	**
np20439	14	1	****	–	–
lm15461	14	1	–	*	**
hk942	14	1	–	–	–
np20613	14	1	*	–	–
np20756	14	1	**	–	–

^*^P = 0.1, **0.05, ***0.01, ****0.005, *****0.001, ******0.0005

^a^Degrees of freedom

### QTL analysis

**Fig. 4:**
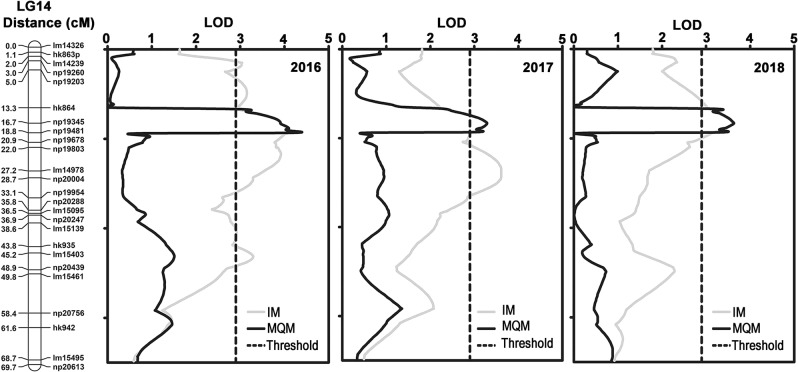
Quantitative traits for *C. gloeosporioides* resistance identified on LG 14. The dotted line indicates the LOD threshold

Based on the parent maps and the integrated map, interval mapping (IM) was used to identify the ripe rot resistance QTLs. In the map of ‘SH’ and the integrated map, a QTL on Chr14 was consistently observed in all three years evaluated. This QTL was named *Cgr1*. In 2016, the maximum LOD score (equal to 4.00) was located at 13.06 cM on Chr14 and explained 19.50% of the phenotype variance. The maximum LOD score was located at 12.06 cM in 2017 and 2018 and explained 16.00% and 17.20% of the phenotypic variance, respectively (Table 4, Fig. 4).

**Table 4:** Summary of the QTL for *C. gloeosporioides* resistance identified on linkage group 14

Map	LG	Year	Mapping type	LOD threshold		LOD_max_ of QTL	LOD max position (cM)	Variance explained (%)
				LG-specific^a^	Genome-wide^a^			
Integrated	14	2016	IM	2.9	3.3	4.00	13.06	19.50
			MQM	2.9	3.3	4.00	13.06	19.50
		2017	IM	3.0	3.1	3.29	12.06	16.00
			MQM	3.0	3.1	3.29	12.06	16.00
		2018	IM	3.0	3.2	3.49	12.06	17.20
			MQM	3.0	3.2	3.49	12.06	17.20
‘Shuang Hong’	14	2016	IM	2.9	3.2	4.00	22.07	19.50
			MQM	2.9	3.2	3.98	15.88	19.40
		2017	IM	2.6	2.9	4.21	26.57	20.00
			MQM	2.6	2.9	3.54	14.88	17.10
		2018	IM	2.6	3.0	3.36	14.88	16.60
			MQM	2.6	3.0	3.36	14.88	16.60

^a^Estimated threshold values using a permutation test with 1000 permutations at *α* = 0.05

To fine-map *Cgr1*, np19345, the nearest marker to the two peak positions, was selected as the cofactor for multiple-QTL mapping (MQM) analysis. For the map of ‘SH’ and the integrated map, the same QTL could also be identified close to np19345 in all three years and explained up to 19.50% of the phenotypic variance (Table 4).

As expected, this QTL could not be identified in the female ‘CS’ map by either IM or MQM methods in any of the three years.

### Correlation between C. gloeosporioides resistance and the np19345 marker

**Fig. 5:**
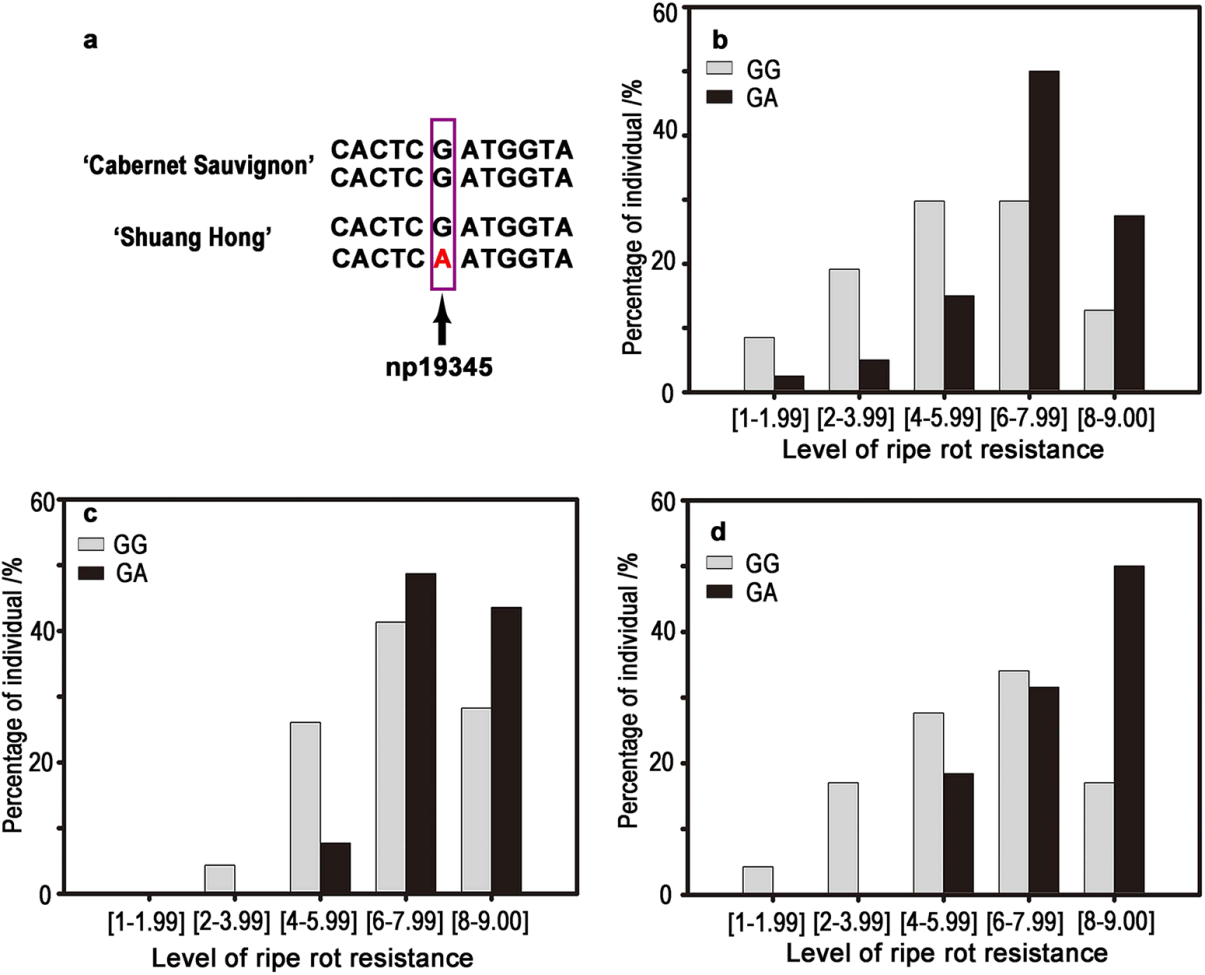
Distributions of *C. gloeosporioides* resistance levels among the hybrids were separated by the presence of ‘GG’ or ‘GA’ alleles at np19345. **a** Base information of the np19345 allele and its flanking sequence; **b**–**d** Ripe rot phenotypes of F_1_ progeny with ‘GG’ and ‘GA’ in 2016 (**b**), 2017 (**c**), and 2018 (**d**)

Np19345, the marker most significantly linked to *C. gloeosporioides* resistance as detected by the Kruskal–Wallis test (Table 3), was also the closest marker to the LOD peak in the *Cgr1* region (Table 4). This marker was located at 4,080,914 bp on Chromosome 14 (Table S1). By analyzing the RAW sequencing data on this marker region, the nucleotides were ‘GG’ in ‘CS’ and ‘GA’ in ‘SH’, respectively (Fig. 5a). The progeny carrying ‘GG’ generally showed susceptible phenotypes, whereas ‘GA’ individuals generally showed resistance, and these results were quite consistent over all three years of disease evaluation (Fig. 5b–d).

### Identification of putative ripe rot resistance genes

**Fig. 6:**
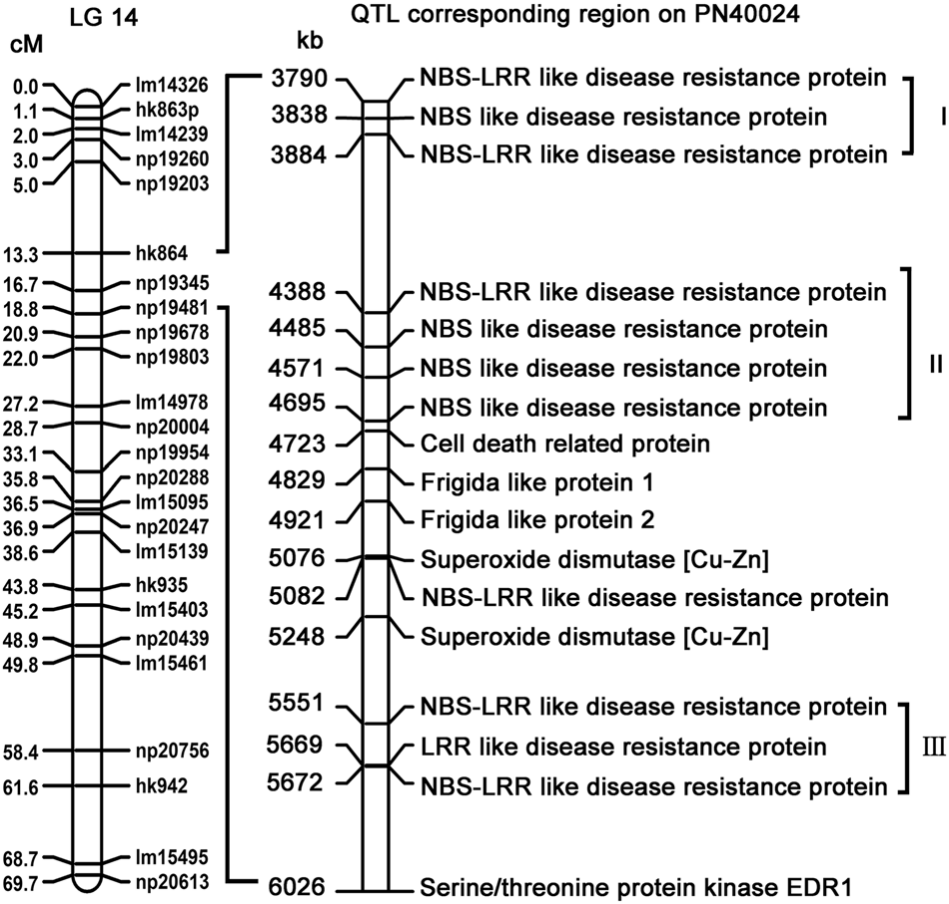
Putative disease resistance genes identified in the grape ripe rot resistance QTL region based on physical locations from the grape reference genome ‘PN40024’

Seventeen biotic/abiotic stress-related genes were identified in the *Cgr1* corresponding region from the grapevine reference genome ‘PN40024’ (Fig. 6). Among these, 11 genes were disease-related ‘R’ genes with NBS and/or LRR domains. Most of these disease-related genes were arranged in three clusters, which contained 3, 4, and 3 genes in regions of 94, 307, and 121 kb, respectively. Between clusters II and III, there was another NBS-LRR-like gene, one cell death-related gene, two frigida-like genes, and two superoxide dismutase [Cu–Zn] genes. Outside cluster III, there was *EDR1*, which was a general disease resistance- and stress tolerance-related gene.

## Discussion

Grape ripe rot caused by the fungal pathogen *C. gloeosporioides* is one of the most serious grapevine diseases. *C. gloeosporioides* can attack different parts of the grapevine tissues, but its main damage is to the ripening berries. The juvenile period of grape hybrids is variable; plants usually take 3-6 years to bear fruit, which makes it difficult to evaluate and select *C. gloeosporioides* resistance using berries in a timely fashion. To establish an in vitro evaluation system for ripe rot resistance in grapevine, Jang et al.^18^ inoculated ripe rot pathogens into several grapevine organs, including young leaves, mature leaves, young stems, and fruits, and they found that young leaves could be used for effectively predicting ripe rot resistance. In this study, we inoculated excised young leaves in three consecutive years and found that ripe rot symptoms appeared consistently. Based on the phenotype data, a QTL for ripe rot resistance was mapped on Chr14 of *V. amurensis* ‘Shuang Hong’. This result demonstrated that young leaves could be used for evaluating grape ripe rot resistance and mapping resistance QTLs.

The ripe rot resistances among the hybrids tended to increase from 2016 to 2018 (Fig. 3). This is likely because ripe rot resistance becomes stronger as the vine becomes older. This phenomenon also resulted in a narrower QTL region mapped by the IM method in 2018 than in 2016 and 2017 (Fig. 4).

To date, first-generation and second-generation molecular markers have been used to construct most grapevine genetic maps. In these maps, the average distance between markers mostly ranged from 4.6 cM to 11.5 cM^19–23^. Zyprian et al.^24^ constructed a linkage map with a combination of SSR and SNP markers, and the average distance between markers was reduced to 2.71 cM. Barba et al.^16^ used SNPs to construct two genetic maps, and the average distances between markers were 1.64 cM and 1.46 cM. In this study, SNPs were used to construct a genetic map with an average distance of 1.81 cM between markers. These results indicate that SNP markers are very frequent in grapevines and could significantly improve marker density compared to that of maps made using first- and second-generation markers.

Resistance (*R*) genes containing conserved domains, such as nucleotide-binding sites (NBS) and leucine-rich repeats (LRR), mainly function in disease resistance in plants^25^. In grapevine, at least 386 putative *R* genes have been predicted^26^, some of which play important roles in downy mildew^27–29^, powdery mildew^30^, and anthracnose^29^ resistance. Many of the disease resistance QTL regions were found to host a number of ‘R’ genes by comparison to the reference genome of the grapevine. For example, the corresponding region of *Rpv10*, a downy mildew resistance QTL identified on Chr9 from ‘Solaris’, contained 26 NBS-LRR genes corresponding to the genome region of ‘PN40024’^13^. The corresponding region of the *Rpv1/Run1* locus, which cosegregated with both downy and powdery mildew, contained 11 NBS-LRR genes, and two of these 11 genes were verified as functional genes in response to the respective diseases^31^. In the present study, according to the ‘PN40024’ reference genome, the *Cgr1* region contained 11 putative disease resistance genes with NBS and/or LRR domains (Fig. 6). Therefore, we believe that one or more of these predicted ‘R’ genes could determine ripe rot resistance in *V. amurensis* ‘Shuang Hong’ grapevine. In the future, we will interest to investigate them further to understand the genetic basis of resistance to grape ripe rot.

## Materials and methods

### Mapping population

The population was obtained by crossing *V. vinifera* ‘Cabernet Sauvignon’ and *V. amurensis* ‘Shuang Hong’ in 2011. Seeds (151) were germinated in a greenhouse in 2012, and seedlings (126) were planted in the greenhouse of the China Agricultural University experimental station, Haidian District, Beijing, China. The true hybrids (91) were confirmed using eight SSR markers (VMC1G3.2, VMC1G7, VMC5H5, VMC8G6, VRZAG83, VVIF52, VVIN56, and VVIP31).

### Disease evaluation

The ripe rot pathogen, *C. gloeosporioides*, was isolated from an infected leaf and confirmed by sequencing *C*. *gloeosporioides* was cultured on potato dextrose agar (PDA) medium at 28 °C in the absence of light until the mycelium spread throughout the medium. After the mycelium was removed, the medium was cultured at 28 °C in the presence of light to promote spore production. The spore suspension was adjusted to ~100,000 spores per mL using sterile distilled water with 0.1% (v/v) Tween-20.

At the ten-leaf stage, which usually occurs in early May, sixteen square pieces (1-cm-side length) were sampled in each year from the fourth and fifth fully expanded leaves of each individual with surgical scissors and were placed on wet filter paper in Petri dishes with the abaxial side up. Leaf pieces were artificially inoculated by spraying with *C. gloeosporioides* suspension and stored in the dark at room temperature. Six days after inoculation, the disease symptoms were scored as 1, 3, 5, 7, or 9 based on the area of necrotic patches (1 = not limited, vast necrotic patches; 3 = numerous necrotic patches; 5 = limited necrotic patches; 7 = less necrotic patches; 9 = punctuated or no necrotic patches) (Fig. 2). The resistance score of each individual was equal to the average score of the 16 leaf pieces. Two leaf pieces from each of the progeny were inoculated with water as a control in each experiment.

### DNA extraction

DNA was extracted from young leaves of the parents and progeny by the hexadecyl trimethyl ammonium bromide method, as described by Qu et al.^32^.

### Genotyping and map construction

Library construction and sequencing were performed using the genotyping-by-sequencing method as described by Elshire et al.^33^ with minor modification. Mse I (New England Biolabs, Ipswich, MA, USA) and HaeII (New England Biolabs, Ipswich, MA, USA) were used to digest the DNA. Two adaptors with 6-nucleotide barcodes were ligated to the digested DNA fragments. By mixing all the samples together, we constructed a DNA pool. Primers complementary to the adaptor sequences were used to amplify the pool. PCR products between 400 and 425 bp were selected from agarose gel. Paired-end sequencing (PE150) was performed for the selected fragments using an Illumina 2500 platform (Illumina, San Diego, CA, USA) by Novogene Bioinformatics Technology Co., Ltd. (Beijing, China). Finally, we identified 7,598 high quality SNPs that could be used to construct a genetic map (Table S1) (unpublished). To avoid markers from the same genetic bin, the segregation type of each marker was analyzed, and only one SNP among markers of the same segregation type was retained. Moreover, we found that, if multiple-QTL mapping (MQM) with cofactor was selected for mapping by MapQTL 6.0 software, 50 markers was the threshold allowing computation in each linkage group for the cross CP population. Therefore, fewer than 50 markers on each chromosome that were distributed uniformly in physical distance were selected to construct the maps.

The CP model of JoinMap 4.0 was used to construct the linkage groups^34^. We selected lm × ll and hk × hk type markers to construct the map of the female parent (CS) and nn × np and hk × hk type markers to construct the map of the male parent (SH). Markers with too many missing genotypes or showing significantly distorted segregation (*P* < 0.001) were discarded. A LOD score equal to seven was the threshold to decide whether loci were linked. To optimize the marker order, markers with *X*^*2* ^> 3.0 were excluded. The Kosambi function was used to calculate the genetic distance between markers. After the parent maps were constructed, the ‘*Combine Groups for Map Integration*’ function was used to construct the integrated map.

### QTL analysis

We used MapQTL 6.0^35^ to calculate marker cosegregation and QTL position. The phenotypic (.qua file), map (.map file), and loci (.loc file) information were imported into MapQTL 6.0. The Kruskal–Wallis algorithm was employed as a nonparametric test to identify markers that were significantly associated with the trait. Interval mapping (IM) was used to detect putative QTLs related to the trait in a 0.5-cM step size. The marker close to the position with the highest LOD in each sampling year was selected as a cofactor. MQM was used for further accurate calculation of the putative QTLs detected by the IM test combined with the cofactor in 0.5-cM steps. The genomic-wide and LG-specific LOD threshold (*α* = 0.05) was calculated by 1000 permutation tests.

## Supplementary information

Table S1 Basic information of single nucleotide polymorphisms (SNPs) identified by the “genotyping-by-sequencing” method
